# Correction to “Structure and Pore Size Distribution in Nanoporous Carbon”

**DOI:** 10.1021/acs.chemmater.2c02766

**Published:** 2022-09-21

**Authors:** Yanzhou Wang, Zheyong Fan, Ping Qian, Tapio Ala-Nissila, Miguel A. Caro

In our recent article,^1^ the given eqs 9–11 for the isotropic projection of stiffness tensors are incorrect. The coefficients multiplying the biaxial elastic constants are too small by a factor of 2. These are the correct expressions (with the corrected coefficients highlighted in bold):
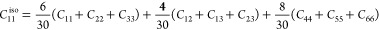
9
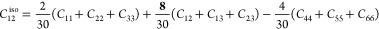
10
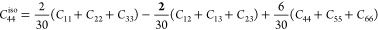
11

This error does not affect the calculated values displayed in tables and figures throughout the paper, which rely on the implementation in the Mattpy code,^2^ which is correct. We thank Jiuyang Shi from Nanjing University for bringing this issue to our attention.
